# Online learning satisfaction and its associated factors among international students in China

**DOI:** 10.3389/fpsyg.2022.916449

**Published:** 2022-07-22

**Authors:** Mei Tian, Genshu Lu

**Affiliations:** ^1^School of Foreign Studies, Xi’an Jiaotong University, Xi’an, China; ^2^School of Humanities and Social Science & West China Higher Education Evaluation Center, Xi’an Jiaotong University, Xi’an, China

**Keywords:** online learning satisfaction, online learning environments, student engagement, international students in China, higher education

## Abstract

The outbreak of the COVID-19 pandemic has caused a substantial transition of Chinese international education to online learning. This article discusses the impact of online learning from international students’ perspectives. Data were collected from exploratory interviews with a small group of international students at a research university and a nationwide survey involving 1,010 international students at 41 universities in China. A synthesis of the two studies’ findings pointed to low levels of online learning satisfaction, particularly among international students from Africa, those in undergraduate programs, those in life sciences and medical disciplines, and those studying at research-centered universities. Moreover, both studies revealed low emotional engagement significantly predicted international students’ online learning dissatisfaction. To enhance international students’ satisfaction, it is suggested that universities and teachers prioritize the building of student-centered online learning environments supporting international students’ emotional involvement and helping them feel a greater sense of belonging in online intercultural learning.

## Introduction

The outbreak of the COVID-19 in December 2019 brought about global disruption, with national borders closed, cities locked down and more than 1.5 billion learners shifting from in-person to online learning (UNESCO, 2020). Before the pandemic, with its economic success and increasing global influence, China had risen as the world’s leading international student host country. By the end of 2018, 492,185 international students from 196 countries were enrolled in 1,004 Chinese higher education institutions (HEIs, Ministry of Education of China, 2019). Previous studies on international students in China have reported on these students’ reasons to choose China as their study destination, their learning experiences and identity formation, and the effectiveness of national policies and international education programs (Tian and Lowe, 2014; Tian et al., 2020).

Since the COVID-19 outbreak, China’s international education has rapidly switched online using various asynchronous and synchronous communication tools. An unpublished estimate of the MoE shows that as of today, approximately 200,000 international students registering with Chinese universities remain outside China and have to rely on online teaching to continue their education. Internationally, scholars have reported students’ reduced satisfaction with and increased disengagement in online education during the pandemic (Chan et al., 2021a). However, few studies have focused on international students in China and examined the impact of COVID-19 from these students’ perspectives, except for several surveys exploring COVID-related depression and psychological distress (Li et al., 2021; Xu, 2021) and an interview study on multilingual experiences (Li et al., 2020).

Drawing on the data generated by a nationwide survey, complemented by the findings of a small-scale exploratory interview study, this article explores international students’ perceptions of emergency remote education, with a focus on their online learning satisfaction and its associated factors. Although located in China, the article contributes to the international discussion of the sustainable and transformative development of “innovative, inclusive, and equitable” online international education (Chan et al., 2021b, p. 7). It holds implications for practitioners and institutions supporting international student learning in the contexts of higher education digitalization and virtual internationalization in the post-pandemic era.

## Literature review

### Student satisfaction in online learning

A large body of research has been conducted on the technological movement of teaching and learning in higher education, often focusing on the teaching, management, and functionality of technology in online learning (Rhode et al., 2017; Rabiman et al., 2020). To better assess online education quality, scholars have called for the research to adopt a “student-centered” approach, investigating attitudinal and affective aspects of online learning and particularly how learners feel content with the courses designed to support their learning (Coates, 2006).

Student satisfaction refers to students’ “subjective experiences” in HEIs and the perceived value of learning experiences (Astin, 1993, p. 273). In classroom-based studies, student satisfaction has been accepted as a valid indicator of institutional performance and higher education quality. Early in the 1980s, Ramsden and Entwistle (1981) identified the relationship between student satisfaction and their quality of learning. Launched in 2005, the UK National Student Survey has been annually conducted on students’ course satisfaction to support public accountability (Officeforstudents.org.uk, 2022).

In the context of online learning, researchers argued that Ramsden and Entwistle’s (1981) model also applies (Johnson, 2006). Drawing on Ramsden and Entwistle (1981), a general theoretical model was proposed by Richardson (2006) that clarified the direct influences of learner demographics, perceptions of online learning environments, and learning behaviors on online learning outcomes. According to this theoretical model, students’ demographic backgrounds directly affect online learning outcomes, perceptions of academic environments directly affect online learning outcomes, and study behaviors directly affect online learning outcomes. Empirical evidence has supported that online learner satisfaction is one of the “five pillars of quality” (Moore, 2005, p. 2), contributing to online course commitment (Nashaat et al., 2021) and learner retention (Lee et al., 2018). In this article, we draw on Richardson (2006) and investigate international students’ online learning satisfaction and the direct effects of demographic background, perceived online learning environments, and engagement in online learning on their satisfaction, facilitating the transformation of emergency remote teaching to sustainable online international education in China and beyond.

### Factors associated with online learning satisfaction

#### Online learning environments

Online learning environments refer to the environment where learning takes place through electronic devices and internet-based technology (Moore et al., 2011). Much research has explored features of online learning environments and their effects on learning, but findings are inconclusive. Wei and Chou (2020), for example, noted that online learning environments were characterized by accessibility, adaptability, interactivity, knowledge acquisition, and ease of loading. Accessibility concerned the provision of access to rich learning resources. Interactivity concerned the opportunities to communicate and interact online with peers and teachers. Knowledge acquisition concerned the contribution of online learning to learners’ expansion of academic knowledge. Adaptability concerned the ability to flexibly manage learning despite temporal and physical limitations (see also Seeletso, 2021). Ease of loading indicated reduced burden and increased ease of learning in online contexts. Stacey and Rice (2002) stressed that the web-based ICT medium enhanced human interaction, breaking down the isolation experienced by physically distanced students. Nevertheless, other researchers reported the negative influence of the online environments on learning, including “…lack of appropriateness for all subjects/course content, increased cost of technological update, program startup costs and challenges, potentially reduced student/professor interaction, irrelevance of previous location advantage, and potential infringement on existing program” (Palvia et al., 2018, p. 236).

Research on conventional classroom learning environments, which has been widely conducted in the USA (e.g., Moos, 1979) and Australia (e.g., Fraser et al., 1982), and later in Africa (e.g., Aldridge et al., 2006) and Asia (e.g., Baek and Choi, 2002), has consistently reported a statistically significant relationship between students’ perceptions of learning environments and their overall satisfaction (e.g., Den Brok et al., 2006). Since the outbreak of COVID-19, researchers have stressed the significant differences between well-planned distance education and the remote courses arising in response to the crisis (Hodges et al., 2020). Studies have reported challenges faced by learners in the pandemic, regarding inequitable internet infrastructure (Tao and Gao, 2022), difficulties in using online platforms (Chen et al., 2020), poorly-prepared online pedagogy (Chan et al., 2021a), growing social distance between students and teachers (Wut and Xu, 2021), and increasing stresses and mental health concerns (Akpinar, 2021), the findings of which highlighted students’ reduced satisfaction with emergency online learning (Chan et al., 2021a). A special issue of the *System* journal presents a recent research exploration of online language education in the pandemic that documented language learners’ difficulties and language teachers’ efforts to support online learning (Tao and Gao, 2022). These studies have provided critical insights into the impact of online learning environment on learner satisfaction in the pandemic. However, few have focused on international students in China. Hence, an investigation into international students’ perceptions of the online learning environment and whether and how their perceptions predict their online learning satisfaction is imperative.

#### Student engagement in online learning

A crucial indicator of effective learning through online education is the successful engagement of students in online academic activities, which is important to prevent dropouts and increase retention of online learners (Bolliger and Halupa, 2018). In higher education, a well-accepted definition of student engagement was framed by Hu and Kuh (2002), i.e., “the time and effort students devote to activities that are empirically linked to desired outcomes of college” (p.555), reflecting the constructionist idea that students act as active agents to construct their knowledge through participation in a supportive learning environment. Research in traditional classroom teaching has consistently established a positive relationship between behavioral participation in academic and extracurricular activities, such as class attendance and disruptive behavior avoidance, concentration, and completion of learning tasks, and increased levels of satisfaction among locals (Pike et al., 2011; Kahu, 2013) and international college students (Korobova and Starobin, 2015). Concerning online learning contexts, Murillo-Zamorano et al. (2019) examined and confirmed the effect of behavioral engagement on student satisfaction. Focusing on flipped classrooms, the researchers found that teaching methods supporting active engagement facilitated students’ understanding of teaching content, which in turn enhanced their satisfaction. Similarly, Fisher et al.’s (2018) study on 348 Australian college students indicated that an engaging, well-designed flipped classroom improved learning performance and increased learner satisfaction.

Recently, researchers have stressed that engagement is a multi-dimensional construct, covering also cognitive and emotional aspects of student learning experiences (Fredricks et al., 2004). Compared to behavioral engagement, the cognitive and emotional dimensions of student engagement are less observable or easily identifiable (*ibid*). The cognitive dimension emphasizes students’ mental investment to complete cognitive and metacognitive tasks and achieve deep learning (*ibid*). The emotional dimension stresses students’ emotional connections with teachers, fellow students, and the learning content and often involves identification with an institution and a feeling of appreciation of positive learning outcomes (*ibid*). Luo et al. (2019) reported that learners’ emotional engagement moderated the effect of cognitive engagement on their satisfaction. Other researchers (Sagayadevan and Jeyaraj, 2012; Dubovi and Tabak, 2021) stressed that greater emotional engagement links to greater behavioral and social engagement, enhancing learning outcomes. A recent study indicated that college students’ emotional engagement had significant and direct influences on their online learning satisfaction in COVID-19 (El-Sayad et al., 2021).

Although often treated as a sub-dimension of behavioral engagement, social engagement has been empirically validated as a separate construct from behavior, cognitive and emotional engagement in online learning environments (Deng et al., 2020; see also Bolliger et al., 2010). Social engagement has been defined as “reciprocal events [in which]…at least two objects and two actions mutually influence one another” (Wagner, 1994, p. 8), involving three types of interaction in online education, i.e., student–student interaction, student-instructor interaction, and student-content interaction (Moore, 1989). Student–student and student-instructor interactions are two-way relationships, while student-content interaction is a one-way process of “intellectually interacting with the content…” (Moore, 1989, p. 2). Previous studies reported the impact of two-way interpersonal communication (Burnett et al., 2007), and student-content interaction (Alqurashi, 2019) on online learning satisfaction. For example, Kuo et al. (2014) reported that learner-content and learner-instructor interactions were the strongest predictors of student online learning satisfaction. Jung et al. (2002) reported that collaborative interaction significantly predicated learner satisfaction in a web-based instruction environment. Despite the previous research, it is not clear whether and how international students’ behavioral, cognitive, emotional, and social engagement predict their satisfaction with the online education provided by Chinese HEIs, making this topic deserving of research attention.

#### Demographic factors

Previous research has investigated how online learning satisfaction varies among student demographic subgroups. The demographic factors possibly affecting learner satisfaction include prior online learning experience, level of education, and country of origin. For example, Jan (2015) reported that students familiar with online teaching modes were likely to feel satisfied with online courses. Simsek et al. (2021) reported that postgraduate students were more satisfied than undergraduates, showing a greater capacity of postgraduate students to adapt to online education. Investigating online experiences of 30,383 students in 62 countries, Aristovnik et al. (2020) reported the highest level of learning satisfaction among students in Oceania, North America, and Europe, followed by those in Asia and South America, while students in Africa reported the lowest levels of satisfaction.

The medium of instruction is regarded as “the most important decision in the internationalization of [Chinese] higher education” (Wan and Gao, 2020, p. 37). In China, college programs are provided to international students in both Chinese and English. The influences of English-medium-instruction (EMI) on student satisfaction have been reported in Asian countries (Peng and Samah, 2006; Kim and Yoon, 2018), and are worth exploring in China in e-learning contexts.

Medicine was among the first EMI programs that China offered to international students and has long been the most popular discipline of study among international students in China (Ministry of Education China, 2019). In the recent decade, with their growing academic strength, engineering disciplines, particularly those at national key “double-first-class” universities, have attracted a continuously increasing number of inward international students and were ranked as the 2nd most popular discipline type in 2018 (Liu, 2018; Ministry of Education China, 2019). Internationally, research results are mixed regarding how student online learning satisfaction is associated with the discipline of study. Several studies reported the disruption of anatomy teaching (Singal et al., 2020), postponement of internship opportunities (Guadix et al., 2020), and cancelation of clinical training (Hilburg et al., 2020), highlighting medical students’ low satisfaction in online learning contexts during the pandemic. Other researchers found that students in sciences and technology tended to be less satisfied than students in health (Aristovnik et al., 2020), as sciences and engineering teaching often contains abstract concepts and complex derivation processes, and hence, is less effective online (Tang et al., 2020). Moreover, Simsek et al. (2021) reported greater satisfaction with the emergency online learning among students in sciences and engineering than those in social sciences and medicine. The inconclusive research findings indicate the need to examine possible disciplinary differences in the satisfaction of China’s inward international students, so as to respond appropriately to further develop online international education.

## Two studies

### Exploratory interview study

An exploratory interview study was designed and conducted as a pilot for the large-scale survey. The interviews broadly explored international students’ online learning experiences. The research questions were listed as follows:

1. How do international students perceive the pandemic-induced emergency online learning?
2. What has influenced international students’ perceptions of emergency online learning?

Interviews were conducted at a national key research-centered university in central China (hereafter referred to as CU). In 2019 CU hosted over 3,000 international students in non-degree, undergraduate, and postgraduate courses in its 24 schools and colleges. On February 5^th^, 2020, the Ministry of Education of China issued the policy of “suspending classes without stopping learning” (Ministry of Education China, 2020). In response to the policy, CU, similar to other Chinese HEIs, requested that all teaching shift online in the spring 2020 semester. The requirement applied to both domestic and international students. In fall 2020, with the normalization of pandemic prevention and control in China, CU re-opened to domestic students. At the time of this manuscript’s preparation in 2022, slightly over 200 international students remain on campus. These students take disciplinary courses in classrooms with Chinese students, while continuing to take required Chinese courses online with the international students who remain outside China.

In April 2021, under the support of CU’s international school, an invitation email was sent to these international students on campus, fully explaining the purpose of the exploratory study. Following the invitation, eight international students volunteered to participate in the interviews without compensation. Table 1 presents the profiles of the interview participants. The small interview sample size allowed few generalizable conclusions, but the in-depth interviews revealed the significant perceptions and the issues affecting their perceptions.

**Table 1:** Interviewees’ profiles.

Student	Level of education	Discipline	Gender	Nationality
S1	Short-term	Chinese	M	Japanese
S2	Undergraduate	Chinese	F	Russian
S3	Undergraduate	Chinese	F	Vietnamese
S4	Postgraduate	Computer science	M	Grenadian
S5	Undergraduate	Chinese	M	Lesotho
S6	Undergraduate	Medicine	M	Moroccan
S7	Postgraduate	Civil engineering	M	Liberian
S8	Postgraduate	Public administration	F	Pakistani

The interviews took the form of informal conversations in which the interviewers encouraged the participants to freely narrate their online learning experiences, allowing in-depth exploration into possible characteristics of international students’ perceptions and issues of concern. The interviews were conducted face to face or *via* online conferencing tools, either in Chinese, English or a mixture of both, based on participants’ preference. Each interview lasted 1 h to 1.5 h. All interviews were automatically transcribed by a digital audio recorder, and the transcripts were checked word by word by the interviewers. Using thematic analysis (Braun and Clarke, 2006), the data were initially coded under the broad categories of “perceptions of emergency online learning” and “factors affecting students’ perceptions of emergency online learning.” As we engaged further with the transcripts, the categories were modified into themes, including “dissatisfaction with online learning,” “perceived online learning environment” factors, and “online learning engagement” factors affecting international students’ online learning satisfaction.

### Survey study

#### Research focus

Based on the interview findings, a nationwide survey was designed to explore international student online learning satisfaction on a much larger scale, aiming to answer the following research questions:

1. What are the characteristics of international students’ perceived online learning satisfaction?
2. Are international students’ perceived online learning environments and engagement correlated with online learning satisfaction? If yes, do international students’ perceived online learning environments and engagement predict their online learning satisfaction?
3. Do international students’ demographic factors, such as country of origin, level of education, medium of instruction, discipline, and type of institution, predict their online learning satisfaction?

#### Procedure and participants

In July 2021, 41 Chinese HEIs located in central, eastern, and western China were approached. Classroom teaching to international students at these institutions has been replaced by remote education *via* various online platforms, such as *TencentMeeting*, *DingTalk*, and *Zoom*. The institutions’ international offices or schools of international students approved the survey. They informed international students of the questionnaire, which was open through *WenJuanXing*, an online survey platform, from July 26^th^ to August 22^nd^, 2021. The research purpose was fully explained, and anonymity and voluntary participation principles were clarified at the beginning of the questionnaire. A total of 1,010 international students participated in the survey. Table 2 presents the survey respondents’ demographics.

**Table 2:** Survey respondents’ profiles.

Category	Frequency	%
Gender		
Male	598	59.2
Female	412	40.8
Total	1010	100.0
Country of origin		
Asia	767	75.9
Africa	161	15.9
Europe, America, and Oceania	42	4.2
Missing	40	4.0
Total	1010	100.0
Medium of Instruction		
Chinese	321	31.8
English	689	68.2
Total	1010	100.0
Prior online learning experiences		
Yes	242	24.0
No	768	76.0
Total	1010	100.0
Discipline		
Arts, humanities, and social sciences	292	28.9
Sciences and engineering	257	25.4
Life sciences and medicine	461	45.6
Total	1,010	100.0
Type of institutions		
Double first-class university	612	60.6
Non-double-first-class university	326	32.3
Missing	72	7.1
Total	1010	100.0
Level of education		
Undergraduate	596	59.0
Postgraduate	394	39.0
Missing	20	2.0
Total	1010	100.0

#### Instruments

The survey adopted the Online Learning Perception Scale (Wei and Chou, 2020) to assess five aspects of the participants’ perceived online learning environments: *accessibility* to various resources, *interactivity* that supported communication, *adaptability* to overcome temporal and spatial limitations, and effectiveness to facilitate *knowledge acquisition* and *ease of loading* (i.e., to reduce learning burden). The survey also adopted the Student Engagement Survey (Burch et al., 2015) to assess the participants’ *behavioral engagement*, *emotional engagement*, *cognitive engagement online,* and *cognitive engagement offline*. Twenty-six items were adopted from Kuo et al. (2014) and Bolliger and Martin (2018) to assess the participants’ *social engagement*, as measured by online student–student, student-instructor, and student-content interactions. Nine items were adopted from Chen and Adesope (2016) and Tseng et al. (2018) to assess the participants’ *satisfaction* with their online learning. All items were measured on a 5-point Likert scale, ranging from “strongly disagree” (1 point) to “strongly agree” (5 points). The original items of the adopted scales were added or deleted based on understandings gained from the exploratory interviews. Original expressions were modified to fit the Chinese context, and the broader national, disciplinary, and linguistic backgrounds of the participants. Two international students studying at CU piloted the questionnaire. Based on their feedback, further changes were made.

#### Data analysis

Researchers have proved the construct validity of the measures in pre-pandemic contexts (Kuo et al., 2014; Burch et al., 2015; Bolliger and Martin, 2018; Wei and Chou, 2020). This research, using AMOS 22.0, performed confirmatory factor analysis (CFA) to test whether the international student participants’ responses fit the measurement models. Representative indices, including the Root Mean Square Error of Approximation (RMSEA), Comparative Fit Index (CFI), and Tucker-Lewis Index (TLI), were used to assess the goodness of fit for CFA. Using SPSS 22.0, the research calculated Cronbach’s α coefficient values to assess the reliability of the scales. Descriptive statistics were computed. Pearson’s correlations were calculated between all variables. Multiple regression analysis was also performed to explore the predictive power of the participants’ demographic characteristics, perceived online learning environment, and online learning engagement for their online learning satisfaction.

## Results

### Study 1: Exploratory interviews

The interview study explored the online learning experiences of a small group of international students at a research university in China. The in-depth interviews revealed the interviewees’ overall dissatisfaction and how this dissatisfaction related to their perceptions of online learning environments and engagement in online learning. Table 3 presents the themes emerging from the interviews, with one or two interview extracts illustrating each theme.

**Table 3:** Interview themes and representative extracts.

Theme	Interview extracts
Perceptions of the emergency online learning	
Value	“*This* [online course] *is good because… they did not delay anything, education, exam, research, lecture.*” (S8)
Dissatisfaction	“*I would not recommend* [the online course]. *All my classmates… hope to return to face-to-face classroom teaching soon*.” (S1) “…*it would not make sense to apply for it, pay the same amount of school fees as if you were on campus and then um continue to use your own resources, your own data, your own space to study.*” (S5)
Factors affecting online learning satisfaction	
Perceived online learning environment	
*Poor internet connectivity*	*“When the weather is not good, for example, when it’s raining, the internet connection gets bad and I would lose signals. I could hear the teacher in the beginning and then I could not hear what they were saying.*”
*Insufficient internet speed in developing countries*	*“Internet speed is a problem in some countries that are not very developed. Even if you buy the best internet, the cables and the infrastructures are old and will bring you this* [slow] *speed of internet.” (S6)*
*Time differences*	*“Here are 5-h differences between Beijing and Russia. No way can I take live classes. I have to watch recorded lectures.”* (S2)
*Distractions at home*	“*…taking online classes at home, it easily gets interrupted. Always someone tells you something or tell you to do something.*”
*Lack of teacher-student interaction*	“In most cases, it is the teacher makes all the talks.”
*Lack of peer interaction*	*“We did not have much contact with our fellow students, we did not know what other students look like…*” (S3)
*Few teaching/learning activities*	“*There are no activities because of the connection issue. It did not work so they stopped.*” (S8)
*Teachers’ incompetency to handle interactive teaching online*	“*In online classes most teachers or if I can say all teachers do not have such abilities… to motivate students to participate* [in interactive learning]” (S6)
Online learning engagement	
*Lecture attendance and assignment submission*	“*Similarly* [with what I did before the pandemic]*, I study roughly 7 h a day,* i.e.*, 4–5 h in* [online] *classes, and another 2–4 h for assignments.*” (S1)
*Reduced attention to teaching*	“*… You know teachers cannot see you. When others are answering questions, it is okay you do not listen. Offline classes are much more effective because you can talk to teachers and other students.*” (S2)
*Difficulty in maintaining focus*	“*I found it difficult to maintain focus*.” (S5)
*Increasing boredom and growing lack of interest*	*“Everyone is behind the camera. How depressing… you open your laptop, sit maybe for 30 min, teachers keep talking and you get bored. You even fall asleep or something …*” (S7)

A brief interpretation of the themes is provided as follows: First, the participants acknowledged that online education allowed them to continue education in this critical situation, but they tended not to perceive that online courses had effectively supported their subject learning. Students questioned the value relative to the financial cost, as online courses provided few opportunities for experiences of hands-on activities or in-person campus life. They were also reluctant that they would recommend their online courses to prospective students:

Second, interviewees expressed dissatisfaction with online learning environments. The participants based in economically less advanced countries reported poor internet connection and a lack of sound infrastructure. Those in home countries having significant time differences with China tended not to perceive that live lectures online were convenient or easily accessible. Those living with families reported online learning distractions at home:

Third, all interviewees expressed high appreciation of teachers’ devotion and commitment to online teaching. However, most of them described their online pedagogy as didactic lecturing, which hardly was satisfying. Some interviewees attributed the lack of interaction to teachers’ incompetency in organizing or managing interactive online teaching. Others referenced poor network quality, which would not allow question answering, feedback, or discussions:

Fourth, the interviewees reported that they managed to attend online lectures and submit assignments on time. They, however, stressed increasing boredom and frequent absent-mindedness, particularly in classes where teachers and classmates turned the camera off to enhance internet connectivity. A growing lack of interest in learning was apparent among these students. The reduced cognitive and emotional engagement negatively affected their satisfaction with emergency online learning.

### Study 2: Nationwide survey

#### Construct validity, reliability, and correlations

Drawing on the findings of study 1, study 2 was conducted to explore the characteristics of and factors associated with international student online learning satisfaction. Using AMOS, the confirmatory factor analysis (CFA) was conducted. As shown in Table 4, the CFA results indicated the acceptable model fit indices, with RMSEA values of less than 0.1 (MacCallum et al., 1996; Kunina-Habenicht et al., 2009), CFI values of 0.9 or above, and TLI values of 0.9 or above (Browne and Cudeck, 1992; Hu and Bentler, 1999), confirming that the scales provided valid measures of the participants’ perceived online learning environments and their engagement in online learning.

**Table 4:** Construct validity.

Measure	*χ* ^2^	df	P	RMSEA	CFI	TLI
Online learning environment	923.984	179	*p* < 0.001	0.064	0.974	0.969
Student engagement (social)	2502.359	296	*p* < 0.001	0.086	0.929	0.922
Student engagement (behavioral, emotional, cognitive online and cognitive offline)	1672.513	161	*p* < 0.001	0.096	0.939	0.928

As shown in Table 5, the Cronbach’s α coefficient values of the perceived online learning environment factors were between 0.936 and 0.964, the Cronbach’s α values of the student engagement factors were between 0.924 and 0.970, and the Cronbach’s α value of online learning satisfaction was 0.973. The results of the reliability analysis indicated a high internal consistency of the measures, with the Cronbach’s α coefficient values of all factors being 0.8 or higher (Pallant, 2020).

**Table 5:** Reliability, correlation matrix, and descriptive statistics.

Factors	Item number	1	2	3	4	5	6	7	8	9	10	11	12	13
1.OLE Accessibility	4	(0.952)												
2.OLE Interactivity	5	0.873^**^	(0.956)											
3.OLE Adaptability	4	0.836^**^	0.859^**^	(0.941)										
4.OLE Knowledge acquisition	4	0.849^**^	0.860^**^	0.888^**^	(0.964)									
5.OLE Ease of loading	4	0.767^**^	0.798^**^	0.833^**^	0.828^**^	(0.936)								
6.SE Student–student interaction	12	0.761^**^	0.777^**^	0.719^**^	0.721^**^	0.664^**^	(0.970)							
7.SE Student-instructor interaction	10	0.769^**^	0.765^**^	0.715^**^	0.716^**^	0.670^**^	0.879^**^	(0.960)						
8.SE Student-content interaction	4	0.821^**^	0.832^**^	0.815^**^	0.843^**^	0.765^**^	0.804^**^	0.831^**^	(0.946)					
9.SE Emotional engagement	6	0.817^**^	0.829^**^	0.807^**^	0.849^**^	0.759^**^	0.753^**^	0.756^**^	0.843^**^	(0.946)				
10.SE Behavioral engagement	6	0.653^**^	0.621^**^	0.617^**^	0.626^**^	0.543^**^	0.658^**^	0.693^**^	0.612^**^	0.711^**^	(0.954)			
11.SE Cognitive engagement online	4	0.719^**^	0.729^**^	0.682^**^	0.694^**^	0.616^**^	0.750^**^	0.756^**^	0.700^**^	0.766^**^	0.835^**^	(0.924)		
12.SE Cognitive engagement offline	4	0.707^**^	0.675^**^	0.659^**^	0.663^**^	0.605^**^	0.726^**^	0.752^**^	0.679^**^	0.715^**^	0.785^**^	0.826^**^	(0.953)	
13.OLS	9	0.818^**^	0.828^**^	0.816^**^	0.845^**^	0.790^**^	0.722^**^	0.724^**^	0.813^**^	0.853^**^	0.604^**^	0.682^**^	0.656^**^	(0.973)
Mean		3.13	2.97	3.02	3.02	2.90	3.14	3.29	3.03	2.97	3.57	3.33	3.48	2.90
Standard Deviation		1.22	1.26	1.25	1.28	1.25	1.12	1.11	1.24	1.23	1.19	1.18	1.22	1.23

** *p* < 0.01. Each factor’s Cronbach’s α coefficient value is presented along the diagonal.

OLE, Online Learning Environment; SE, Student Engagement; OLS, Online Learning Satisfaction.

Table 5 also presents the correlation matrix for the factors of online learning environments, student engagement, and online learning satisfaction. The analysis revealed a strong positive correlation between satisfaction and the five environmental factors (*r* > 0.7, Illowsky and Dean, 2018), and a moderate to a strong positive correlation between satisfaction and the seven engagement factors (0.7 > *r* > 0.2, *ibid*). A higher level of the participants’ perceived online learning environment strongly correlated with a higher level of satisfaction, while a higher level of the participants’ engagement in online learning strongly to moderately correlated with a higher level of satisfaction.

#### Descriptive statistics

Using SPSS 22.0, study 2 computed descriptive statistics. As shown in Table 5, the survey participants perceived online learning environments less favorably than online learning engagement, while their online learning satisfaction received the least favorable mean scores. Specifically, the mean scores of the five online learning environment factors ranged from 2.90 to 3.13, with interactivity (M = 2.97, SD = 1.26) and ease of loading (M = 2.90, SD = 1.25) scoring lower than the median value (3). The seven engagement factors received a mean score between 2.97 to 3.57, with behavioral engagement scoring the highest (M = 3.57, SD = 1.19) and emotional engagement scoring the lowest (M = 2.97, SD = 1.23).

Moreover, the mean score of online learning satisfaction was 2.90, worse than the median value (3). As presented in Table 6, 21.31% of the participants strongly disagreed that they were satisfied with online teaching activities, 22.82% strongly disagreed that they were satisfied with online learning content or course structure, 27.46% strongly disagreed that online courses well served their educational needs, and 32.67% would not recommend online courses to others.

**Table 6:** Means of online learning satisfaction.

Items	Strongly disagree	Disagree	Neutral	Agree	Strongly agree	Mean
1. I feel that my online course serves my needs well	27.46%	4.58%	8.03%	17.99%	11.93%	2.72
2. I would recommend my online course to other students	32.67%	2.97%	25.38%	17.52%	11.46%	2.62
3. I am satisfied with my teachers’ teaching methods	18.75%	11.74%	27.46%	25.57%	16.48%	3.09
4. I am satisfied with the learning content and structure of my online course	22.82%	12.88%	27.37%	21.97%	14.96%	2.93
5. I am satisfied with my teachers’ teaching styles	18.84%	12.12%	27.27%	26.61%	15.15%	3.07
6. I am satisfied with the discussion and activities	21.31%	12.97%	28.22%	23.58%	13.92%	2.96
7. I am satisfied with the assignments and the criteria of the assignments	20.17%	10.42%	28.79%	25.85%	14.77%	3.05
8. I am satisfied with the tests and exams	19.98%	10.7%	28.22%	24.15%	16.95%	3.07
9. Overall, I am satisfied with my online learning experiences	28.22%	11.74%	23.96%	22.82%	13.26%	2.81

#### Multiple regression: Factors associated with online learning satisfaction

Study 2 performed multiple regression analysis to examine the impact of demographic factors, student engagement factors, and online learning environment factors on online learning satisfaction. The analysis involves two phases. In the first phase, the research analyzed how demographic factors, student engagement factors, and online learning environment factors, respectively, affected online learning satisfaction. The purpose was to compare the explanatory power of these three groups of factors for international students’ online learning. In the second phase, the research simultaneously analyzed the impact of demographic factors, student engagement factors, and online learning environment factors on online learning satisfaction. The purpose was to identify the factors with statistically significant influences on online learning satisfaction.

As presented in Table 7, models 1, 2, and 3, respectively, examined the impact of demographics, student engagement, and online learning environment on online learning satisfaction. As shown by the *R^2^* values of the three models, online learning environment (77.4%) was the strongest predictor of the variation of online learning satisfaction, followed by online engagement (76.1%) and demographic factors (11.2%). Model 4 simultaneously examined the impact of demographic factors, student engagement, and online learning environments on satisfaction, explaining the variation of online learning satisfaction by 81.6%. However, compared to model 2 and model 3, the *R^2^* value of model 4 was only slightly increased, indicating possible interaction among demographic factors, student engagement factors, and online learning environment factors.

**Table 7:** Factors associated with online learning satisfaction.

Independent variable	Dependent variable: Online course satisfaction			
	Model 1 *β* (Std error)	Model 2 *β* (Std error)	Model 1 *β* (Std error)	Model 4 *β* (Std error)
Country of origin (Europe, America, and Oceania as reference)				
Asia	−0.027 (0.205)			−0.050 (0.095)
Africa	0.022 (0.221)			−0.068^*^ (0.103)
Level of education (Postgraduate as reference)	−0.172^***^ (0.092)			−0.048^**^ (0.043)
Medium of instruction (English as reference)	0.093^*^ (0.099)			0.006 (0.046)
Prior online learning experience (No prior experience as reference)	0.166^***^ (0.098)			0.001 (0.046)
Discipline (Life sciences and medicine as reference)				
Arts, humanities, and social sciences	0.153^***^ (0.118)			0.055^**^ (0.055)
Sciences and engineering	0.150^***^ (0.116)			0.042^*^ (0.054)
Institution type (Non-double-first-class university as reference)	−0.137^***^ (0.098)			−0.053^**^ (0.046)
Student–student interaction		0.063 (0.038)		0.014 (0.035)
Student-instructor interaction		0.011 (0.042)		0.015 (0.039)
Student-content interaction		0.267^***^ (0.035)		0.063 (0.036)
Emotional engagement		0.570^***^ (0.033)		0.366^**^ (0.034)
Behavioral engagement		−0.070^*^ (0.031)		−0.049 (0.029)
Cognitive engagement online		0.017 (0.037)		−0.017 (0.034)
Cognitive engagement offline		0.054 (0.031)		0.027 (0.028)
Accessibility			0.198^***^ (0.034)	0.120^***^ (0.034)
Interactivity			0.199^***^ (0.036)	0.100^**^ (0.036)
Adaptability			0.065 (0.037)	0.034 (0.036)
Knowledge acquisition			0.307^***^ (0.037)	0.142^***^ (0.038)
Ease of loading			0.170^***^ (0.029)	0.131^***^ (0.028)
*F*	15.970^***^	455.735^***^	689.226	201.268^***^
*R^2^*	0.122	0.761	0.774	0.816

* *p* < 0.05;

** *p* < 0.01 and

*** *p* < 0.001.

*The standard errors of the regression coefficients are given in the brackets*.

As shown by model 4, concerning demographics, participants from Africa reported significantly lower levels of satisfaction than those from Europe, America, or Oceania. Undergraduate participants reported significantly lower levels of satisfaction than postgraduate participants. Respondents studying at national elite double-first-class universities reported significantly lower levels of satisfaction than those studying at non-double-first-class universities. Respondents in AHS and SciE disciplines reported higher levels of satisfaction than those in life sciences and medicine (LMed). Medium of instruction and prior online learning experiences did not exert a significant impact on satisfaction. Concerning online learning environment factors, accessibility, interactivity, knowledge acquisition, and ease of loading had significant positive influences on satisfaction. Concerning student engagement factors, emotional engagement had significant positive influences on satisfaction. It is worth noting that among all factors, emotional engagement had the highest standardized regression coefficient and hence the strongest predictive power for international students’ satisfaction with emergency online learning.

## Discussion

### Characteristics of international student online learning satisfaction

Both studies pointed to important features of international students’ online learning satisfaction during COVID-19. The exploratory interview study revealed international students’ dissatisfaction with unsatisfactory internet quality and connectivity, time zone differences, and distractions when learning online at home. The interviews also pointed to the lack of peer interaction, inadequacy of didactic lecturing, and ineffective support of cooperative online learning. The findings of the limited student–student and student-lecturer interaction are congruent with the results of earlier studies (e.g., Wut and Xu, 2021).

Based on the findings of the exploratory interviews, the survey study was designed and conducted, focusing on a much larger group of international students. The descriptive statistics indicated low levels of online learning satisfaction among the survey respondents. The mean value of online learning satisfaction was lower than the mean values of perceived online learning environments or those of student engagement. The least positive responses were regarding the students’ willingness to recommend the online course to others.

Of the online learning environment factors, interactivity and ease of loading scored least favorably, indicating that survey respondents perceived that online learning environments tended not to facilitate interactions or effectively reduce learning burden or pressure. The results align with previous research findings on domestic students’ experiences of emergency remote education (Wut and Xu, 2021). Moreover, of the seven engagement factors, emotional engagement scored the lowest, showing that the participants were unlikely to feel excited or energetic when online learning. Pre-pandemic research has reported linguistic, social, and cultural challenges encountered by international students in intercultural learning (Tian and Lowe, 2014). Such difficulties can be exacerbated online, which may result in immediate emotional reactions, such as boredom and low interest, and affect senses of being and belonging (Kahu, 2013).

### Demographic factors associated with international student satisfaction

Our interviews focused on a small group of international students at a research-centered university. The survey study examined the online learning satisfaction of international students with broader disciplinary, institutional, and national backgrounds. The results contribute to the literature on how international students’ online learning satisfaction varies by demographics. Specifically, the survey indicated that international students from Africa were less satisfied than those from Europe, America, or Oceanian countries. Scholars have warned of the growing digital divide exacerbating “economic and structural inequalities…among historically vulnerable populations” (Chan et al., 2021a, p. 4). Our results further evidenced the need to attend to African students’ low satisfaction and its antecedents to ensure more equitable online learning.

In addition, the survey results, consistent with the previous studies (Simsek et al., 2021), indicated that education level was significantly associated with online learning satisfaction. Compared to undergraduate participants, postgraduate international students were more satisfied. The reasons may be that in Chinese HEIs undergraduate programs involve much longer teaching hours than postgraduate programs and that postgraduate students have higher time-management, self-motivation, cognitive, and metacognitive skills that may help them better manage online learning challenges (*ibid*).

Moreover, although no statistically significant differences were identified between international students in sciences and engineering disciplines and those in LMed, the survey respondents in Arts, humanities, and social sciences (AHS) reported significantly higher levels of satisfaction than those in life sciences and medicine (LMed). The results were in line with the research findings on the concerns of medical training during the COVID-lockdown (Guadix et al., 2020; Hilburg et al., 2020; Singal et al., 2020). The postponement or cancelation of clinical training (*ibid*) may have affected the professional development of international medical students in China, explaining the low satisfaction among the LMed respondents in this research.

It is worth noting that international students at national elite research-centered double-first-class universities were less satisfied than those at teaching-oriented universities. An earlier survey reported no significant differences between double-first-class and non-double-first-class universities in terms of engaging international students in classroom learning, opposite to the common assumption that elite universities would better support learning (Tian et al., 2020). This research further evidenced that Chinese research universities, despite their generous funding resources to achieve academic excellence (Liu, 2018), did not perform better than teaching universities concerning the provision of satisfactory online international student education.

In contrast to the research findings of Jan (2015), this survey found that prior online learning experience had no significant impact on international student satisfaction, reflecting the great differences between well-designed distance courses and the emergency online education, to the latter of which few prior experiences may be applicable. Moreover, the result that the medium of instruction was independent of satisfaction is different from previous research (Peng and Samah, 2006; Kim and Yoon, 2018). Increasing internationalization has driven the use of EMI in Chinese HEIs. Future research could combine qualitative and survey methods to further examine the online learning satisfaction of international students in EMI courses in Chinese HEIs.

### Learning environment factors associated with international student satisfaction

In our survey study, correlation analysis showed a strong positive correlation between international students’ online learning satisfaction and their perceived online learning environments. Multiple regression analysis showed that international students’ perceived online learning environments contributed the most to their online learning satisfaction, followed by online engagement factors and demographics. Congruent with previous studies (Wei and Chou, 2020), the regression results revealed the four environmental factors having significant effects on satisfaction, i.e., accessibility, interactivity, knowledge acquisition, and ease of loading. In contrast, the analysis found no significant association between adaptability and satisfaction. Flexibility has long been acknowledged as one crucial benefit of online education (Seeletso, 2021). Technical problems, including improper internet facilities and poor internet quality, may have undermined desirable learning outcomes among international students. Besides, the participants with time zone differences had to rely on asynchronous instructions and recorded lectures, which may further reduce their real-time interactions with teachers and fellow students, cause unexpected stresses, and affect their learning satisfaction.

### Student engagement factors associated with international student satisfaction

Previous research has reported a significantly positive association between student engagement and online learning satisfaction (Bolliger and Halupa, 2018). The correlation analysis of our survey showed a moderate to a strong positive correlation between satisfaction and the engagement factors. The multiple regression analysis contributes to the literature, distinguishing emotional engagement as the most important engagement factor predicting the online learning satisfaction of international students in China. Their positive attitudes toward online learning acted as a significant contributor to high levels of satisfaction, while a low aspiration for online learning led to low levels of satisfaction. The result is in line with our interview findings, which revealed the interviewees’ frequent absent-mindedness and reduced learning interest. Low emotional engagement often accompanies feelings of isolation and alienation. Although maintaining enthusiasm in learning is demanding for all students in virtual contexts, it is particularly difficult for international students, who have to simultaneously cope with academic, linguistic, and (inter-)cultural challenges.

The regression results presented no significant relationship between online satisfaction and the three forms of social engagement, i.e., learner-instructor, learner-learner, and learner-content interactions. The results are contrary to the findings of Kuo et al. (2014) or Jung et al. (2002). One possible explanation lies in the very limited interaction between international students and their teachers or classmates, and the consequently affected interactions with teaching content in online courses.

## Conclusion

The COVID-19 pandemic has presented unprecedented challenges to Chinese international education, resulting in a rapid transition from onsite to online learning. This article focuses on international students’ online learning experiences. The discussion was based on the findings of an exploratory interview study with a small group of international students at a research university and the results of a nationwide survey involving 1,010 international students at 41 Chinese HEIs. The interview study functions as the pilot for the survey study, informing the design and conduction of the latter. A synthesis of the two studies’ findings revealed important characteristics of international students’ online learning satisfaction and the factors associated with their satisfaction.

The following limitations exist for the research. First, the two studies were designed and conducted 1 year after the outbreak of the pandemic. The findings may not reflect international students’ perceptions at different stages of the pandemic. Second, the survey study was administered online *via* a Chinese online survey platform, which may have excluded potential participants with internet access difficulties. In addition, although the CFA analysis suggested the acceptable construct validity of the survey scales, the RMSEA values were between 0.064 and 0.096, all higher than 0.050, indicating that the measurement models can be further optimized in future research. Moreover, our survey study applied multiple regression to analyze the direct impact of demographic characteristics, online learning environment, and engagement on online learning satisfaction. We acknowledge that interaction effects may exist between demographic characteristics, online learning environment, and engagement. Future research can take the interrelationships among the predictors into consideration in modeling online course satisfaction.

This research is one of the first attempts to discuss China’s emergency online education from the perspectives of international students. With online learning becoming “a new normal,” the findings hold implications for practitioners and institutions striving for sustainable development of international education in the post-pandemic era. First, it is of importance that a student-centered approach is adopted in the delivery and evaluation of online courses, with the priority given to the building of a student-centered online learning environment. Given the four environmental factors significantly predicting learner satisfaction, student-centered online learning environments should ensure international students’ access to rich learning materials, promote effective interactions with peers and teachers, support acquisition of subject knowledge and expansion of academic capacity, and effectively reduce online learning burden and pressure. While all Chinese institutions are expected to enhance positive online learning environments, national elite double-first-class universities should play a leading role in this endeavor, given the funding, resources, and academic advantages they enjoy.

Significantly, both studies revealed low emotional engagement among international students, which significantly predicated their dissatisfaction. Greater emotional engagement enhances behavior, cognitive, and social engagement, supporting improved learning satisfaction. Hence, we suggest universities and faculty members work together to create and sustain a positive learning climate, which cultivates international students’ interest in and passion for learning while promoting international students’ emotional involvement in online courses. A sense of community and belongingness can be formed and maintained where international students, in navigating their online journeys of intercultural learning throughout this difficult time, perceive that faculty and institutions well understand their concerns and difficulties and are ready to provide technical, academic, social, and emotional support.

## Data availability statement

The original contributions presented in the study are included in the article/supplementary material, further inquiries can be directed to the corresponding authors.

## Ethics statement

Ethical review and approval was not required for the study on human participants in accordance with the local legislation and institutional requirements. The patients/participants provided their written informed consent to participate in this study.

## Author contributions

MT and GL contributed equally to the design of the research, the collection, analysis, or interpretation of the research data, and the writing of the paper. All authors have read and agreed to the published version of the manuscript.

## Funding

The research is partially supported by the National Natural Science Foundation of China (71804145), Shaanxi Province Undergraduate and Further Education Teaching Reform Research Project (21ZG004, 21ZZ003), Higher Education Academy of China “Higher Education Opening Up” Research Project (21ZSYZZD02), and Shaanxi Social Science Research Fund (2018Q03).

## Conflict of interest

The authors declare that the research was conducted in the absence of any commercial or financial relationships that could be construed as a potential conflict of interest.

## Publisher’s note

All claims expressed in this article are solely those of the authors and do not necessarily represent those of their affiliated organizations, or those of the publisher, the editors and the reviewers. Any product that may be evaluated in this article, or claim that may be made by its manufacturer, is not guaranteed or endorsed by the publisher.
